# Red Blood cell distribution width: an emerging diagnostic factor of acute appendicitis?

**DOI:** 10.1186/1749-7922-8-54

**Published:** 2013-12-26

**Authors:** Ergenekon Karagöz, Alpaslan Tanoglu

**Affiliations:** 1 Department of Infectious Diseases and Clinical Microbiology, GATA Haydarpasa Training Hospital, 34668, Uskudar, Istanbul, Turkey; 2 Department of Internal Medicine, Gastroenterology, GATA Haydarpasa Training Hospital, Istanbul, Turkey

## Abstract

Acute appendicitis is the most common surgical abdominal emergency. Immidiate diagnosis of this disease is crucial, because this condition can lead to appendiceal perforation, potential peritonitis, and even death. We read with great interest the article ‘The role of red cell distribution width (RDW) in the diagnosis of acute appendicitis: a retrospective case-controlled study’ by Narci et al. and wanted to discuss whether RDW alone provide certain information about the inflammatory status of the patient with acute appendicitis.

## Dear editor

We read with great interest the article ‘The role of red cell distribution width in the diagnosis of acute appendicitis: a retrospective case-controlled study’ by Narci et al. [1]. They aimed to evaluate whether red cell distribution width (RDW) has a role in the diagnosis of acute appendicitis. The authors concluded that if compared to healthy controls, RDW levels were lower in patients with acute appendicitis. Being inexpensive and easy attainability of this parameter may strengthen its utilization in daily practice in the near future. We would like to thank the authors for their contribution.

RDW which is used in the differential diagnosis of anemia, is an automated measure of the variability of red blood cell size [2]. Previously it was shown that, RDW is an independent variable of prognosis in patients with cardiovascular diseases such as heart failure, myocardial infarction, strokes, and pulmonary hypertension [2-6]. In addition, it was also found to be related to mortality and other severe adverse outcomes in renal and infectious diseases [7]. Aging, malnutrition, Iron or vitamin B12 deficiency, bone marrow depression, or chronic inflammation may affect RDW levels [1,2]. Thus, it would have been better, if the authors had mentioned these RDW affecting factors.

In a previous study, two novel biomarkers, calprotectin (CP) and serum amyloid A (SAA) were found to be related to acute appendicitis [8]. Recent studies have demonstrated that Neutrophil-to-Lymphocyte Ratio and mean platelet volume (MPV) are also associated with inflammatory diseases [9,10]. In this view, it would also be relevant, if the authors included these parameters in the study.

We are of the opinion that the findings of Narci et al. [1] will lead to further research concerning the relationship between RDW and acute appendicitis. Nevertheless, RDW should be considered with other inflammatory markers (e.g. C-reactive protein, procalcitonin, calprotectin) to provide certain information about the inflammatory status of the patient.

## Competing interests

We have no competing interests to declare.
